# Mesothelioma-Associated Fibroblasts Modulate the Response of Mesothelioma Patient-Derived Organoids to Chemotherapy via Interleukin-6

**DOI:** 10.3390/ijms25105355

**Published:** 2024-05-14

**Authors:** Mario Cioce, Veronica Gatti, Fabiana Napolitano, Noemi Maria Giorgiano, Andrea Marra, Giuseppe Portella, Alfonso Fiorelli, Francesca Pentimalli, Vito Michele Fazio

**Affiliations:** 1 Laboratory of Molecular Medicine and Biotechnology, Department of Medicine, University of Campus-Biomedico of Rome, 00128 Rome, Italy; 2 Institute of Translational Pharmacology, National Research Council of Italy (CNR), 00133 Rome, Italy; 3 Department of Translational Medical Sciences, University of Naples Federico II, 81025 Naples, Italy; 4 Thoracic Surgery Unity, Department of Translational Medical Sciences, University of Campania Luigi Vanvitelli, 80138 Naples, Italy; 5 Department of Medicine and Surgery, LUM University, 70010 Casamassima, Italy

**Keywords:** mesothelioma patient-derived organoids, PDO, IL-6, mesothelioma-associated fibroblasts, cocultures, cisplatin, pemetrexed, chemoresistance

## Abstract

Malignant pleural mesothelioma (MPM) remains an incurable disease. This is partly due to the lack of experimental models that fully recapitulate the complexity and heterogeneity of MPM, a major challenge for therapeutic management of the disease. In addition, the contribution of the MPM microenvironment is relevant for the adaptive response to therapy. We established mesothelioma patient-derived organoid (mPDO) cultures from MPM pleural effusions and tested their response to pemetrexed and cisplatin. We aimed to evaluate the contribution of mesothelioma-associated fibroblasts (MAFs) to the response to pemetrexed and cisplatin (P+C). Organoid cultures were obtained from eight MPM patients using specific growth media and conditions to expand pleural effusion-derived cells. Flow cytometry was used to verify the similarity of the organoid cultures to the original samples. MAFs were isolated and co-cultured with mPDOs, and the addition of MAFs reduced the sensitivity of mPDOs to P+C. Organoid formation and expression of cancer stem cell markers such as ABCG2, NANOG, and CD44 were altered by conditioned media from treated MAFs. We identified IL-6 as the major contributor to the attenuated response to chemotherapy. IL-6 secretion by MAFs is correlated with increased resistance of mPDOs to pemetrexed and cisplatin.

## 1. Introduction

MPM is an aggressive cancer characterised by a high clinical latency and is often diagnosed at an advanced stage when curative surgery is not possible [1,2]. Histologically, MPM can be classified as epithelioid (the most common subtype, accounting for 60–80% of cases), sarcomatoid (~10%), and biphasic (10–15%) [1]. Since 2003, the frontline treatment for unresectable MPM has been platinum+pemetrexed chemotherapy [3]. More recently, the USFDA has approved the immune checkpoint inhibitor nivolumab+ipilimumab, which shows a significant but limited survival benefit [4,5]. Many different classes of agents with different mechanisms of action have been tested with rather disappointing results [6]. This appears to be due to the very high resistance of MPM cells to anticancer agents [7]. In addition, therapy-induced stress remodels the MPM environment. We and others have shown that profound rearrangements occur in MPM cultures under stress-induced chemotherapy [8]. These include genomic, epigenetic, proteomic, metabolic, and secretomic changes, all of which accelerate intra-tumour heterogeneity and the emergence of progenitor-like, EMT-driven, chemoresistant cell subpopulations [8,9,10]. To further complicate this scenario, preclinical studies have often been performed in models that partially fail to recapitulate the complexity of the disease. The development of patient-derived organoids (PDOs) has emerged as a promising tool in this regard. PDOs are three-dimensional, self-organising structures of cancer cells isolated from surgical specimens or biological fluids [11]. PDOs have been shown to recapitulate the cytoarchitecture and, to a significant extent, the heterogeneity of the tumour of origin [12]. Indeed, PDOs can accurately represent the genomic landscape of their source in terms of mutation rates, DNA methylation patterns, gene expression signatures, and copy number variations [13]. This makes PDOs clinically relevant tools for disease modelling towards predictive drug screening [14,15]. Cocultures of PDOs with components of the tumour microenvironment (TME) (i.e., cancer-associated fibroblasts, CAFs) can provide important information, as CAFs represent a large cell subpopulation of the TME that is actively involved in tumour progression/tumour resistance [16]. For example, cytokines released by CAFs exposed to 5-FU induce pro-tumorigenic changes in colorectal cancer organoid-derived cells [17]. Recently, mesothelioma-associated fibroblasts (MAFs) have been isolated and shown to express markers that partially overlap with CAFs from other tumours [18,19,20]. Here, we have successfully established mPDO cultures from eight MPM patients using pleural effusion-derived cells. Primary MPM samples and serially passaged (p3) mPDOs were cytologically similar with respect to the expression of mesothelial markers (MSLN; CALB2; KRT5/6; PDPN). As expected in a clinically relevant model, the size and number of mPDOs were altered in a patient-specific manner when pharmacologically relevant doses of chemotherapeutic agents were added. We derived MAFs to co-culture with the mPDOs. The addition of MAFs altered the response of mPDOs to pemetrexed + cisplatin towards increased resistance. Conditioned medium from chemotherapy-treated MAFs raised the expression of cancer stem cell markers, including ABCG2 and CD44, in the recipient mPDOs. We found increased levels of IL-6 in the supernatant of the treated co-cultures and demonstrated that this was derived from the MAFs. This increased secretion of IL-6 by MAFs was functionally relevant because pretreatment of conditioned media with IL-6-neutralising antibodies strongly attenuated the response of mPDOs to chemotherapy. 

## 2. Results

**Figure 1:**
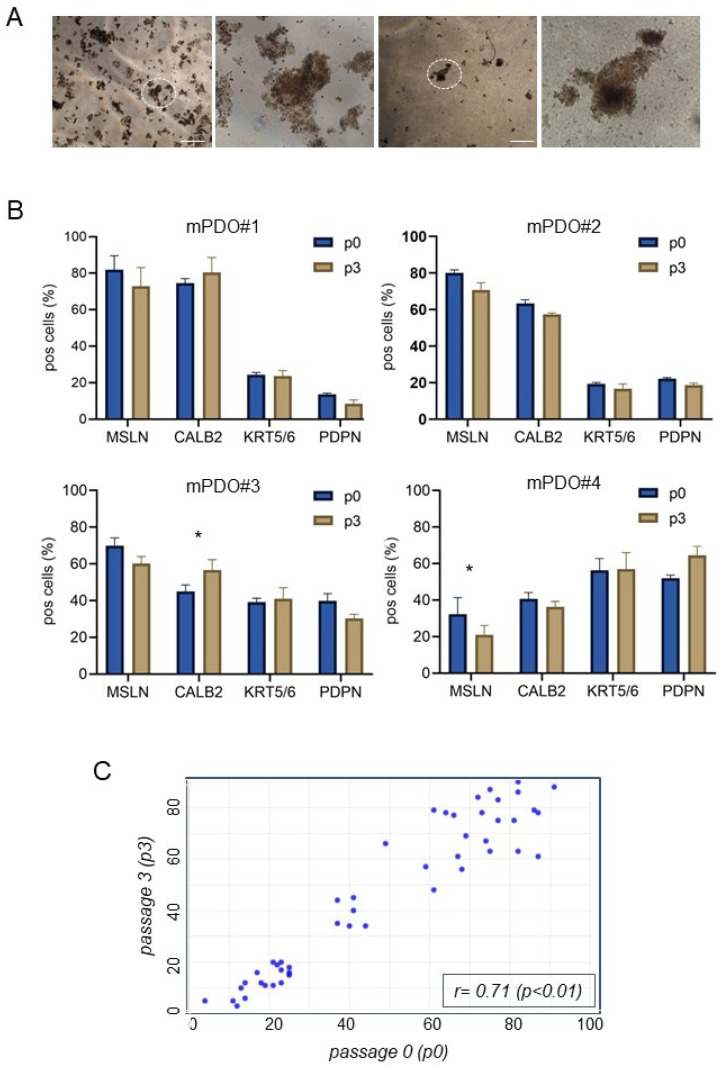
Validation of mesothelioma patient-derived organoid (mPDO) cultures. (**A**) Representative micrographs of passage two organoids formed from pleural effusions of malignant pleural mesothelioma patients, as described in Section 4. Size bar: 200 μm. Magnification of the circled PDO is shown to the right of each panel. (**B**) Flow cytometry analysis of cells expressing mesothelin (MSLN), calretinin (CALB2), keratin 5/6 (KRT5/6), and podoplanin (PDPN) at passage 0 (immediately after Dispase II treatment, p0) and after three passages (p3). (**C**) Scatter plot of p0 vs. p3. Percentage of marker expressing cells from mPDOs in (**B**) at passage 3 (y-axis) and those at passage 0 (x-axis) were reported in the graph (triplicate experiments). Linear correlation analysis results reported in the figure. Statistics: * *p* < 0.05; note that differences between p0 and p3 expression of markers were not significant except where indicated.

Establishment of mPDO cultures. We established mPDOs (n = 8) from pleural effusion-derived cells. The medium composition was defined starting from a basal medium, with the addition of specific factors that proved to be key for the propagation of the mPDOs (see Section 2 and Section 3, please). The establishment rate for this small cohort of samples was 66% (8/12), and the propagation rate up to passage 5 was 60%. The majority of mPDOs exhibited irregular morphology with clusters of mesothelial cells of varying complexity (Figure 1A). In addition, the timing of organoid formation was variable, ranging from several hours to several days (range: 14 h–5 days). 

We demonstrated that primary MPM samples and passaged mPDOs were cytologically similar. In fact, the obtained mPDOs expressed mesothelial markers (including mesothelin, calretinin, cytokeratin 5/6, and podoplanin) when serially passaged (p3), very similar to the original sample, immediately after thawing and disaggregation (p0) (Figure 1B). A high correlation was observed between the percentage of positive cells (for all mesothelial markers) at passage 0 and the percentage of positive cells at passage 3 (r = 0.71; *p* < 0.01). (Figure 1C). Thus, the percentage of cells expressing these markers was maintained and persisted over time and passages.

**Figure 2:**
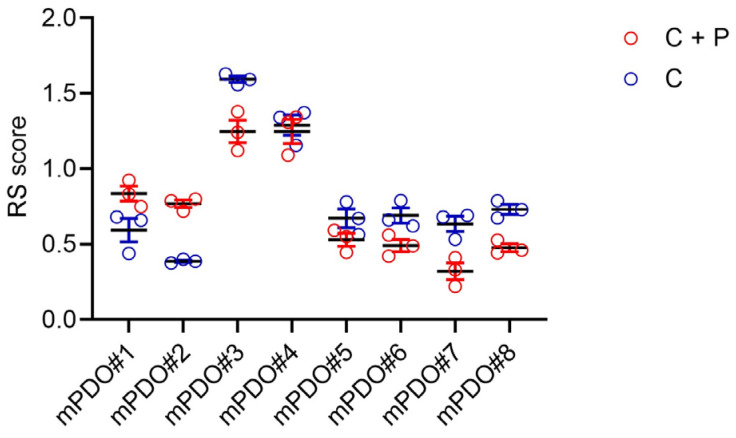
A large proportion of mPDOs were resistant to cisplatin + pemetrexed (C+P) treatment. Representative graphs of eight mPDO cultures treated with cisplatin (2 ugr/mL) (C) or cisplatin + pemetrexed (213 ng/mL) (C+P) for 96 h (with drug washout after 24 h). The effect of the drug was assessed by an empirically defined response score (RS) according to the formula: number of organoids formed × mean maximum diameter at day 0/number of organoids × mean maximum diameter at day 4. An RS ≤ 1 indicates resistance to treatment. Mean + SE of triplicate experiments.

Response of mPDOs to pemetrexed + cisplatin. Next, we evaluated the response of the mPDOs cisplatin (2 ugr/mL) (C) or to cisplatin + pemetrexed (213 ng/mL) (C+P) for 96 h (with drug washout at 24 h). When challenged with C or C+P, mPDO cultures responded heterogeneously, as expected in a clinically relevant setting. We measured the effect of the treatment using a response score (RS), considering the number of organoids formed, mean diameter, and number of live cells after treatment. The size and number of mPDOs were altered in a patient-specific manner (Figure 2), with six out of eight mPDO cultures resistant to C+P, thus showing an RS ≤ 1 (except for mPDO#3 and mPDO#4). In most cases (except for mPDO#5), we observed a relatively stronger effect of the C+P combination compared to C alone (Figure 2). We selected four of the mPDOs belonging to the C+P resistant subgroup to study their resistance to therapy and its modulation. 

**Figure 3:**
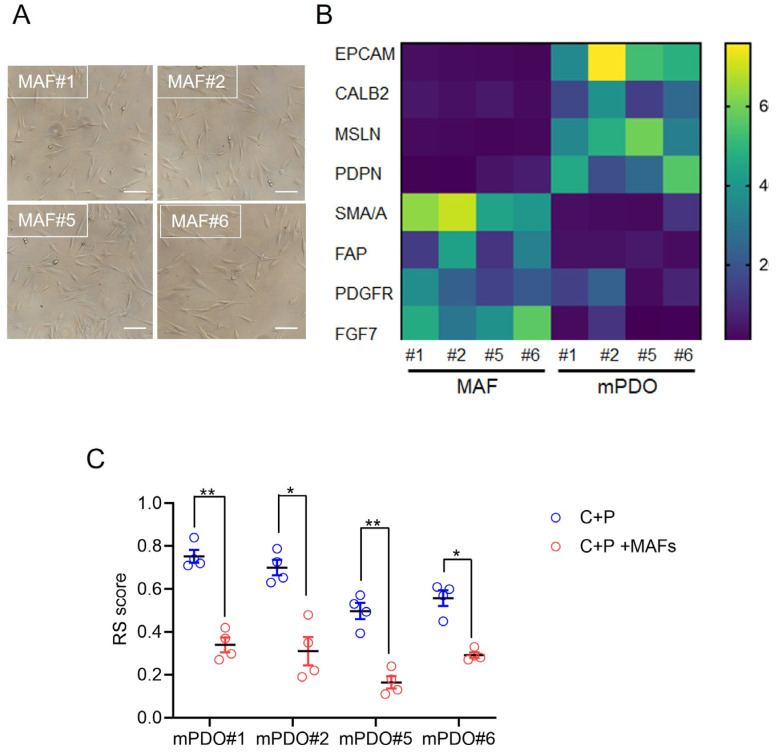
Co-culturing of mPDOs with matched MAFs increased resistance to C+P treatment. (**A**) Representative micrographs of MAFs isolated and propagated as described in the methods. Size bar: 50 μm. (**B**) Mesothelioma-associated fibroblasts (MAFs) did not express mesothelial markers. MAFs were analysed for the expression of mesothelial markers and known cancer-associated fibroblast markers by qRT-PCR. The mean of triplicate experiments is expressed as fold over control (FOC). (**C**) Briefly, passage 3 mPDO#1-2-5-6 showing decreased sensitivity to C+P treatment were treated with C+P for 96 h as single cultures or as co-cultures with MAFs as indicated in the methods (1:1 ratio). RS score is reported. Mean + SE of quadruplicate experiments. Statistics: * *p* < 0.05; ** *p*< 0.01.

Establishment of mesothelioma-associated fibroblast (MAF) cultures. We investigated the effect of co-culturing organoids and matched MAFs on the response to C+P. First, MAFs were derived from the same sample used to obtain PDOs (Figure 3A), and the relative enrichment of the cultures obtained was validated by qRT-PCR at passage 2. Compared to the mPDOs, the MAF cultures were negative for the expression of mesothelin, calretinin, and podoplanin at the mRNA level (Figure 3B). The MAFs were also negative for the expression of EpCAM mRNA, while showing variable but significantly higher expression levels of PDGFR, FAP, SMA/A, and FGF7 (Figure 3B). Thus, MAF cultures showed expression of genes typical of CAFs and negligible expression of mesothelial markers. 

Effect of mPDO + MAF co-culturing on C+P resistance. When challenged with C+P, the mPDO+MAF cocultures showed a different response compared to the mPDOs alone (Figure 3C), in that an increased resistance (higher RS) was observed for all four cocultures tested (Figure 3C). Specifically, the response score was generally lower when compared to the mPDOs cultured alone (Figure 3C, compared to Figure 2). 

**Figure 4:**
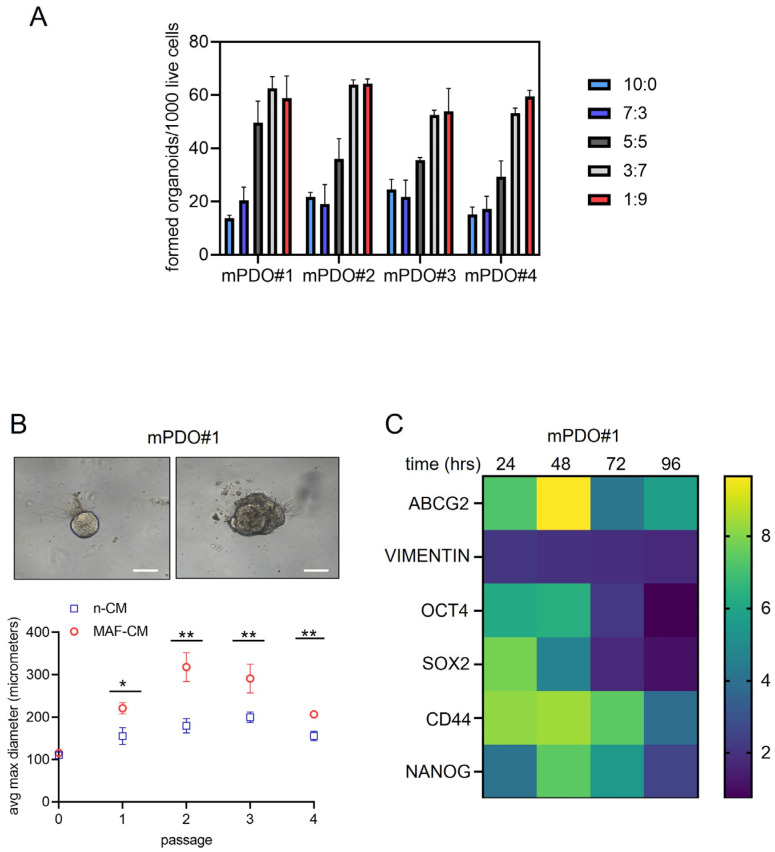
The conditioned medium (CM) of MAFs altered the organoid-forming ability and the size of the formed mPDOs. (**A**,**B**) Organoid-forming ability was recorded after culturing the recipient mPDOs with different ratios of organoid growth medium (OGM) to MAF-conditioned medium (MAF-CM) (medium obtained 24 h after C+P washout). (**B**) Upper panel. A representative image of mPDO#1 treated at passage 3 with either unconditioned OGM medium (n-CM) or MAF-CM. Size bar: 200 μm. Bottom panel: graph showing the effect of MAF-CM on the mean maximum diameter of mPDO#1 at each passage (0–4). Note that passage 0 refers to 5 days after seeding. (**C**) MAF-CM increased the expression of stem cell markers. A representative heat map of the expression levels of the indicated mRNAs, assessed by qRT-PCR, at the indicated time after the addition of MAF-CM. Values are expressed as fold over ctrl (n-CM treated mPDO#1). The mean of two independent experiments is reported. Statistics: * *p* < 0.05; ** *p* < 0.01.

Conditioned medium from C+P treated MAFs affected the number and size of recipient mPDO organoids. To understand how coculturing of mPDOs and MAFs could contribute to a lower RS score after C+P treatment, conditioned medium (CM) was collected after P+C treatment of MAFs and added to a different ratio (of CM to non-CM) to the recipient mPDOs. We found that the organoid-forming ability was dose-dependently increased by the MAF-conditioned medium, with a maximum being reached at the 3:7 ratio (Figure 4A) for three out of the four cultures. Similarly, when passage two mPDOs were treated with MAF-CM, we found a significant effect on their size (Figure 4B, top panel). Specifically, MAF-CM consistently increased the size of the treated mPDOs across passages (Figure 4B, bottom panel, and Supplementary Figure S1). 

MAF-CM modulated the expression of cancer stem cell markers and chemoresistance genes. Organoid forming ability (OFA) is thought to result from the activation of pluripotent, tissue-resident stem cells [21]. This may be associated with the upregulation of stem cell markers. Therefore, we assessed by qRT-PCR whether MAF-CM could increase the expression of cancer stem cell markers in recipient mPDOs (Figure 4C). This showed an early increase in OCT4, SOX2, and CD44, while NANOG increased later and persisted over time. We also evaluated the expression of vimentin, a gene product involved in epithelial-to-mesenchymal transition. We tested the levels of ABCG2 mRNA, as this latter gene product has been implicated in mediating cisplatin resistance [22]. ABCG2 showed an early increase like OCT4, ABCG2, and CD44 after treatment with MAF-CM, whereas vimentin was only slightly increased by MAF-CM (Figure 4C). Such a pattern was variably observed for the remaining three mPDOs (Supplementary Figure S2). Taken together, we found that conditioned media from C+P-treated MAFs could affect OFA and the size of mPDOs, and this correlated with modulation of cancer stem cell markers and chemoresistance genes.

**Figure 5:**
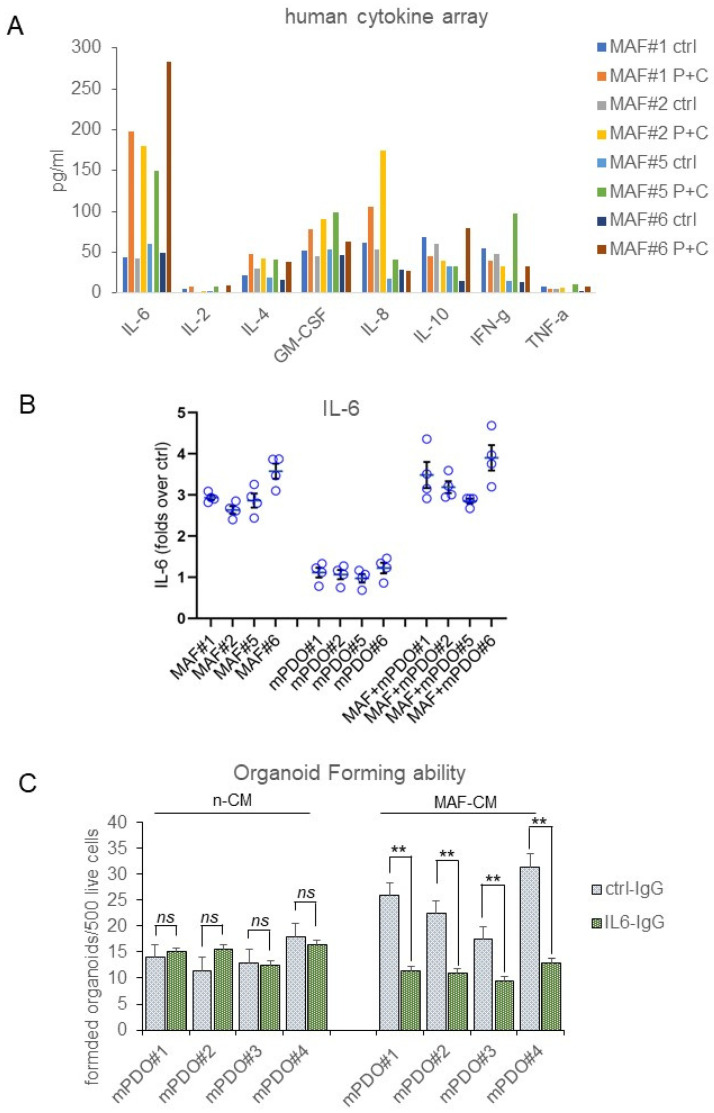
IL-6 was increased in the medium of mPDO + MAF co-cultures after C+P challenge and mediated increased OFA. (**A**) A Luminex-based assay for eight cytokines was used to detect inflammatory chemokines in the conditioned media of mPDO+MAF co-cultures. The histogram shows the average levels of the indicated cytokines from two independent experiments. (**B**) IL-6 is mainly secreted by MAFs after C+P. mPDOs, MAFs, and co-cultured mPDO+MAFs were assayed for IL-6 secretion 24 h after the C+P challenge by indirect ELISA. Results are expressed as fold-over controls, where controls are ctrl (vehicle)-treated cells. (**C**) Increased IL-6 may mediate the resistance of mPDOs to C+P. Y axis: OFA score (number of organoids formed/1000 live cells) was calculated in mPDO cultures challenged with ctrl (MAF-CM, not treated with C+P) or with MAF-CM (treated with C+P), in the presence of anti-IL-6 neutralising antibody (IL-6-IgG) or a control antibody (ctrl-IgG). Histograms represent the mean + SE of three independent experiments. Statistics: ** *p* < 0.01; ns = not significant.

Interleukin-6 released by C+P-treated MAFs contributed to the chemoresistance of mPDOs. To deepen the previous observations, we evaluated the composition of media conditioned by mPDOs + MAFs for 24 h after the C+P challenge. We evaluated cytokine levels using an 8-plex human cytokine array. Interleukin-6 (IL-6) stood out as the most consistently elevated cytokine in all cocultures after C+P treatment (Figure 5A). IL-6 was therefore further investigated for its known role in mediating resistance to chemotherapy in MPM and other cancers [23,24]. We measured the amount of secreted IL-6 in the conditioned medium of mPDOs and MAFs either single or co-cultured at steady state and after P=C treatment by ELISA assay (Figure 5B). This first showed a steady increase of this cytokine from 24 h after treatment (Figure 5B) in both MAFs and mPDO co-cultures. Very little IL-6 was secreted by passage three mPDOs (consistent with the fact that mPDOs at p3 are devoid of TME components) compared to MAFs. Notably, the levels of IL-6 secreted were higher in the co-cultures than in the MAFs, suggesting a paracrine contribution of mPDO-derived cells in stimulating IL-6 release by the MAFs (see Section 3). Taken together, this indicated that MAFs released IL-6 after C+P treatment. 

To assess the functional relevance of the released IL-6, we used an IL-6 inhibitory antibody (Figure 5C). We found that, when compared to its inactive ctrl IgG, the addition of lL-6 neutralising antibody (10 ugr/mL) strongly attenuated the effect of MAF-CM on the OFA (Figure 5C). MAF-CM collected in the absence of chemotherapy treatment did not significantly affect the OFA score of mPDOs (Figure 5C), consistent with the observation that IL-6 was released from MAFs after the C+P challenge (Figure 5B). Taken together, this evokes an important role for IL-6 secreted by MAFs in mediating the response of co-cultures to the chemotherapeutic agents.

## 3. Discussion

Tackling MPM resistance to therapy by identifying targetable determinants of resistance down to the “single patient” level appears to be more feasible thanks to the establishment of patient-derived organoids [25]. Here, we have applied organoid technology to the study of MPM resistance to cisplatin and pemetrexed.

We were not the first to attempt the generation and expansion of mPDOs [26]. Pleural mesothelioma organoids from two patients were established by Mazzocchi and colleagues using a microfluidic device and conventional media [27]. In addition, some important examples from peritoneal mesothelioma have recently been published [25]. We found that the protocol we set up here shows similarities to that published for peritoneal mesothelioma organoids [25], with some key differences. Specifically, we found that the inclusion of FGF9 could significantly increase the yield and stability of the mPDOs generated. FGF9 is prognostic for overall survival in MPM patients (Supplementary Figure S3). We also observed a very important contribution from the use of conditioned medium in previous passages. We believe that one possibility to explain such an effect of conditioned medium is that its retention during passaging may provide a factor secreted by stromal TME cells that is lost during serial passaging since organoid formation is intrinsically biased towards the formation of enriched epithelial structures [28,29]. Another important novelty in our protocol is the addition of low doses of arachidonic acid (ARA). In addition to ARA being a polyunsaturated fatty acid essential for normal health, we and others have shown that arachidonic acid may confer survival properties to MPM cells [30]. It is also a prognostic factor in ovarian cancer ascites [31]. Arachidonic acid is produced by TME components such as endothelial cells, monocytes, and platelets in the absence of stressors [32]. We believe that the addition of ARA at low doses may indeed suffice for the absence of TME-producing cell factors, which in turn may be lost during mPDO passaging. Finally, the doses of ARA used in the OGM medium formulated here are compatible with those found in many human tissues under physiological conditions [33]. We challenged our mPDO with the currently approved first-line chemotherapy regimen for MPM (cisplatin-pemetrexed combination) and obtained a heterogeneous response. We believe this is expected from an experimental model of clinical relevance such as PDOs. Here, we have identified mesothelioma-associated fibroblasts as a determinant of chemoresistance. Our evidence adds to a long history of the relationship between the presence of CAF (MAF) and tumour progression [18,20]. In obtaining MAF cultures, we did not examine the full repertoire of MAF expression markers. However, we found that the MAFs expressed SMA/A, FGF7, FAP, and PDGFR, which is partially consistent with recently published work [18]. We found heterogeneous expression of these markers, which correlates with the demonstrated heterogeneity of such cell subpopulations [34,35,36]. We were able to obtain eight mPDO cultures from twelve pleural effusions. One sample was technically lost, and three did not form organoids. All eight mPDO cultures were derived from epithelioid MPM, so it is possible that the conditions we set up here favor the proliferation of epithelioid MPM lineages. In addition, where time to death after diagnosis was available, we did not observe an association of this parameter with organoid formation propensity, possibly due to the small number of patients. However, the small size of our casuistry (only one biphasic MPM among the three non-PDO-forming MPMs) does not allow us to draw conclusions, and further studies on biphasic and possibly sarcomatoid MPMs are warranted.

IL-6 is produced by many cell types, including endothelial cells, macrophages, epithelial cells, monocytes, and fibroblasts [37]. We found that in MAFs, IL-6 levels were induced by C+P treatment. This is consistent with what has recently been published on the crosstalk between mesothelioma cells and lung fibroblasts, with the former being able to modulate the activation state of lung fibroblasts [19]. In addition, we found that the amount of IL-6 produced when mPDO-derived cells were co-cultured with MAFs was significantly higher than when the MAFs were cultured alone. This may reveal a more complex mechanism of action whereby factors produced by mPDOs may facilitate IL-6 secretion by MAFs under the C+P challenge. A possible candidate would be IL-1 alpha [38]. For example, IL-1α activates the IL-6/IL-8 cytokine network through both NF-κB and C/EBPβ during oncogenic SASP [8,39]. The limited cytokine array did not allow us to investigate this latter possibility, which will be the subject of further investigation in the future.

An interesting question is: what is downstream of IL-6 action on OFA in the mPDOs? We speculate that MAF-derived IL-6 contributes to the adaptive resistance of MPM to therapy by activating the NFkB and STAT3 pathways. 

We and others have shown, both in MPM and in other cancers, that modulation of the number and activity of chemoresistant ALDHbright cells in CRC may be mediated by IL-6-stimulated STAT3 [17]. Another possibility, which is not mutually exclusive, is that NFkB activation may follow IL-6 binding to mPDO-derived cells. This would be consistent with our previous observations that a dual STAT3 and NFKB inhibitor, butein, can attenuate MPM resistance to pemetrexed and cisplatin both in vitro and in vivo [40,41]. A limitation of this study is the number of samples used and, within the eight mPDO cultures established, the selection of those showing resistance to C+P. This may have biased our investigation towards stress-activated mechanisms. We plan to expand our mPDO bank. Another limitation is that we did not investigate whether other stimuli, different in nature from pemetrexed and cisplatin, could induce a similar increase in IL-6. This broader possibility is currently being investigated in our laboratory. 

## 4. Materials and Methods

Source of MPM specimens. MPM pleural exudates and their corresponding cell pellets (n = 12) were obtained from Mesobank, a Research Ethics Committee approved Research Tissue Bank. All the MPM (n = 12) were of mainly epithelioid histology, except for one biphasic histology. The age of the patients ranged from 69 to 93 years. All patients provided written informed consent, and samples were anonymized before they were released. Mesobank is supported by Asthma and Lung UK, the Victor Dahdaleh Foundation, and the June Hancock Mesothelioma Research Fund.

Reagents. Pemetrexed and Cisplatin were from Sellekchem (Houston, TX, USA).

Flow cytometry. mPDOs were mechanically and enzymatically freed of BME, disaggregated, and filtered through a 70 um filter mesh before staining. The following antibodies were employed in separate tubes, each antibody matched to its isotype-specific-related control antibody, in PBS1X-0.2% BSA, for 45 min at 4 °C, light-protected. For the KRT5/6 and CALB2 staining, cell permeabilization was performed before staining with the Cell Fixation & Cell Permeabilization Kit (ThermoFisher, Waltham, MA, USA). For viability assay, the disaggregated PDOs were stained with Sytox Blue Helix NP Blue (Biolegend, CA, USA) for 5 min on ice before flow cytometry. Data were acquired with the CytoFLEX Flow Cytometer (Beckman Coulter, IN, USA) and analysed with CytExpert software (version 2.4.0.28). The following antibodies were employed, from Abcam (Cambridge, UK) and ThermoFisher (Waltham, MA, USA): ABCAM ab315357 Alexa Fluor^®^ 488 Anti-Mesothelin antibody rabbit monoclonal; ABCAM ab303715 Alexa Fluor^®^ 647 Anti-Podoplanin antibody rabbit monoclonal; ABCAM ab210633 PE Anti-Calretinin antibody rabbit monoclonal; ThermoFisher PA5-116450; Cytokeratin 5/6 Antibody (PA5-116450) rabbit polyclonal.

PDO cultures. Mesothelioma patient-derived organoids (mPDOs) cultures were obtained as follows: Cell aggregates were collected from the pleural effusions of diagnosed patients after mild centrifugation (300× g × 10 min at RT). Cell aggregates were washed three times in washing medium: Advanced DMEM-F12 (Thermofisher, Waltham, MA, USA), 0.2% BSA, Amphotericin B, Ciprofloxacin 2 ugr/mL, and insulin 1 ugr/mL and then resuspended in Dispase II solution (Stem Cell Technology, Vancouver, CA, USA) for 15 min at 37 °C in slow agitation. After that, the cells were filtered through a 100 uM strainer (CORNING, NY, USA) and resuspended in organoid growing medium (OGM) composed of Advanced DMEM-F12, 0.5% BSA, 50 ng/mL EGF, 10 ng/mL FGF2, 10 ng/mL FGF9 (Cedarlane Labs, Burlington, CA, USA), 1X B27 (Thermofisher, Waltham, MA, USA), Insulin 10 ugr/mL (St. Louis, MO, USA), recombinant human R-Spondin 1 50 ng/mL (R&D, Minneapolis, MN, USA) and recombinant human Noggin 50 ng/mL (Thermofisher, Waltham, MA, USA), and arachidonic acid (ARA) 1 microMol/L and seeded for 24 h in a BIOFLOAT™ 24 well plate (FaCellitate, Mannheim, Germany) at a density of 5000 cells per well. After 24 h, cells were collected by mild centrifugation without further filtering and resuspended in BME (R&D Systems, Minneapolis, MN, USA) drops (500 live cells/35 uL drop) with a cell-pellet-to-BME ratio of 1:4 on ice and plated in a preheated 24 well dish. After 30 min in the 37C incubator, warm OGM was added to the side of the dish (to avoid dislodging the BME drops) and mixed with 30% of the conditioned medium from the previous pre-aggregation step. 

Validation of PDO cultures. Flow cytometry was performed on both MPM specimens and matched passage 3 mPDOs immediately after mechanical and enzymatic disaggregation. Staining for mesothelin (MSLN), cytokeratin 57 (CK5/7), Podoplanin (PDPN), and Calretinin (CALB2) was performed as described (see before).

Cytokine array. Briefly, PDO culture supernatants were analysed for cytokine production with a Bio-Plex Pro Human Cytokine 8-plex Assay (BIORAD Hercules, CA 94547, USA), according to the manufacturer’s instructions. Plates were read using a Luminex-200 plate reader, and MFIs were normalized to absolute values with the provided standard curve as per the manufacturer’s instructions. A minimum of 6 × 24 wells (containing one BME drop each) were collected for each assay.

mPDO Treatment. mPDO (or mPDO + MAFs) was mechanically and enzymatically disaggregated into single cells, and 500–1000 live cells were plated into BME drops in 24 well plates 24 h before starting treatments. mPDO cultures were treated with cisplatin (2 ugr/mL) (C) or with cisplatin + pemetrexed (213 ng/mL) (C+P) for 96 h (with drug washout at 24 h). We classified the PDOs as resistant or sensitive based on an empirically defined response score (RS), according to the formula: number of formed organoids × average max diameter × viable cells (%) at time 0 day/number of organoids × average max diameter × viable cells (%) after 96 h. Please note that an RS score of 1 denoted no effect, and a RS ≤ 1 indicated resistance to the treatment.

Mesothelioma Associated Fibroblasts (MAFs) isolation and propagation. MAFs were isolated as previously described [17], with some modifications. Briefly, one-third of the MPM cells obtained from the pleural effusions after centrifugation were cultured in plastic dishes in 20% human serum containing OGM for 72 h to enrich for adherent cell subpopulations. After that, the growth medium was shifted to a 20% human-serum-containing advanced DMEM-F12 supplemented with non-essential-aminoacids (NEAA) (ThermoFisher, Waltham, MA, USA), and cells in suspension were removed at each passage by PBS 1X washing. MAFs samples were then tested positive for mRNA expression of fibroblast markers and negative for the expression of mesothelial markers, as indicated within passage five of the isolation. MAFs + PDO cocultures. Disaggregated mPDO-derived cells were mixed in a variable ratio (1:1 to 1:5 live cells) with CAFs and included in BME drops, as previously described, in complete OGM, to start treatment 24 h later. Harvesting of conditioned media. MAFs were treated with either vehicle (Ctrl) or C+P for 24 h, and then media were substituted with OGM for an additional 24 h to allow conditioning of the medium. Conditioned media were collected and centrifuged at 3000 rpm for 10 min before being used or cryopreserved (−80 °C). We did not record significant proliferative effects on the growth of the MAFs when grown with OGM for 24 h to allow conditioning of the medium. 

Detection of IL-6 by ELISA. The amount of IL-6 secreted in the medium of PDO cultures was quantified with the Human IL-6 Quantikine ELISA Kit (R&D, Minneapolis, MN, USA). PDO and mPDO + MAF culture supernatants were centrifuged at 4C and diluted appropriately before detection.

Treatment with IL-6 antibody. Neutralizing monoclonal antibody against human interleukin 6 and its biologically inactive isotype ctrl ab Mouse IgG1, kappa was from Invivogen (Invivogen, San Diego, CA, USA) and was used as indicated in the main text.

mRNA extraction and expression analysis. For RNA extraction, organoid-containing BME drops were collected and mechanically disaggregated on ice. The pellet was washed twice with ice-cold PBS1X and then resuspended in an RNA extraction reagent. Please note that no enzymatic disaggregation of the organoids was performed when subsequently extracting the RNA. Total RNA was extracted using the Trizol Reagent (Thermofisher, Waltham, MA, USA).

qRT-PCR analysis. The first-strand cDNA was synthesized according to the manufacturer’s instructions (M-MLV-RT kit, Thermofisher, Waltham, MA, USA). Gene expression was measured by real-time PCR using the Sybr Green assay (Thermofisher, Waltham, MA, USA) on a 7900HT instrument from Applied Biosystems. All the primers used were from Origene (Rockwille, MA, USA), and sequences will be made available upon request.

Statistics. GraphPad Prism (Version 9.0) was used to perform the data analysis. The data, except where indicated, were from at least three independent experiments and were presented as mean ± SEM. Kaplan Meier analysis was performed with the Xena platform [42].

## 5. Conclusions

We have obtained mPDOs from pleural effusion of MPM patients and shown that, as often seen in clinical settings, their response to pemetrexed and cisplatin is heterogeneous. When analyzing the effect of coculturing MAFs with mPDOs we have found that IL-6 secretion by MAFs, elicited by P+C treatment, correlated with increased resistance to therapy. 

## Data Availability

Any source or data deemed as needed is available upon request.

## Supplementary Materials

The following supporting information can be downloaded at: https://www.mdpi.com/article/10.3390/ijms25105355/s1.
