# Supplementary material for: Mesothelioma-Associated Fibroblasts Modulate the Response of Mesothelioma Patient-Derived Organoids to Chemotherapy via Interleukin-6
Source: Int J Mol Sci. 2024 May 14;25(10):5355. doi: 10.3390/ijms25105355 (PMC11121414; doi:10.3390/ijms25105355)
Supplement: Supplementary file 1 [file ijms-25-05355-s001.zip › Legends to Suppl. Figures.pdf]

Suppl. Figure S1. Graph representing the effect of MAF-CM on the average max diameter of the mPDO#2,3,4 at each passage (0–4). Please note that passage 0 refers to 5 days after seeding.

Suppl. Figure S2. The CM from chemotherapy treated MAFs increased the expression of stem cell markers. Representative heat maps of the expression levels of the indicated mRNAs, as assessed by qRT-PCR, at the indicated time, after addition of the MAF-CM. Values expressed as fold over ctrl (n-CM treated mPDO#2,3,4). The average of two independent experiments is reported. Statistics.

Suppl. Figure S3. Kaplan-Meier plot of 84 MPM assessed for the levels of FGF9 mRNA. Kaplan Meier analysis was performed with the Xena platform [42].
