# Supplementary figures and images for: Mesothelioma-Associated Fibroblasts Modulate the Response of Mesothelioma Patient-Derived Organoids to Chemotherapy via Interleukin-6
Source: Int J Mol Sci. 2024 May 14;25(10):5355. doi: 10.3390/ijms25105355 (PMC11121414; doi:10.3390/ijms25105355)

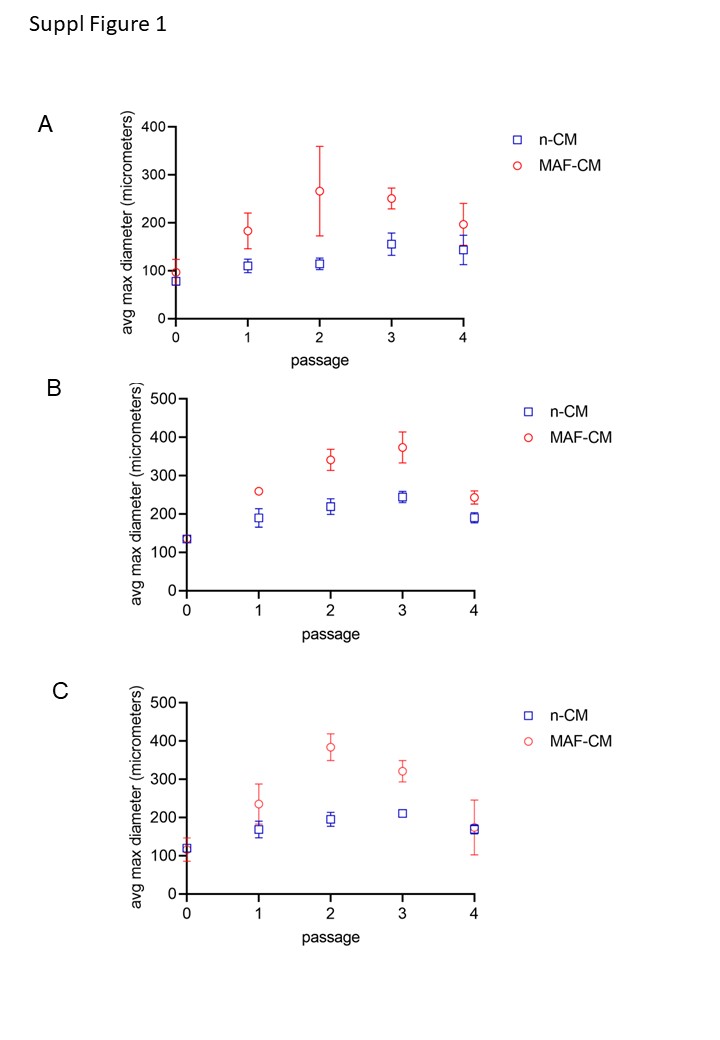

Supplement: Supplementary file 1 [file ijms-25-05355-s001.zip › Suppl Fig.1.JPG]

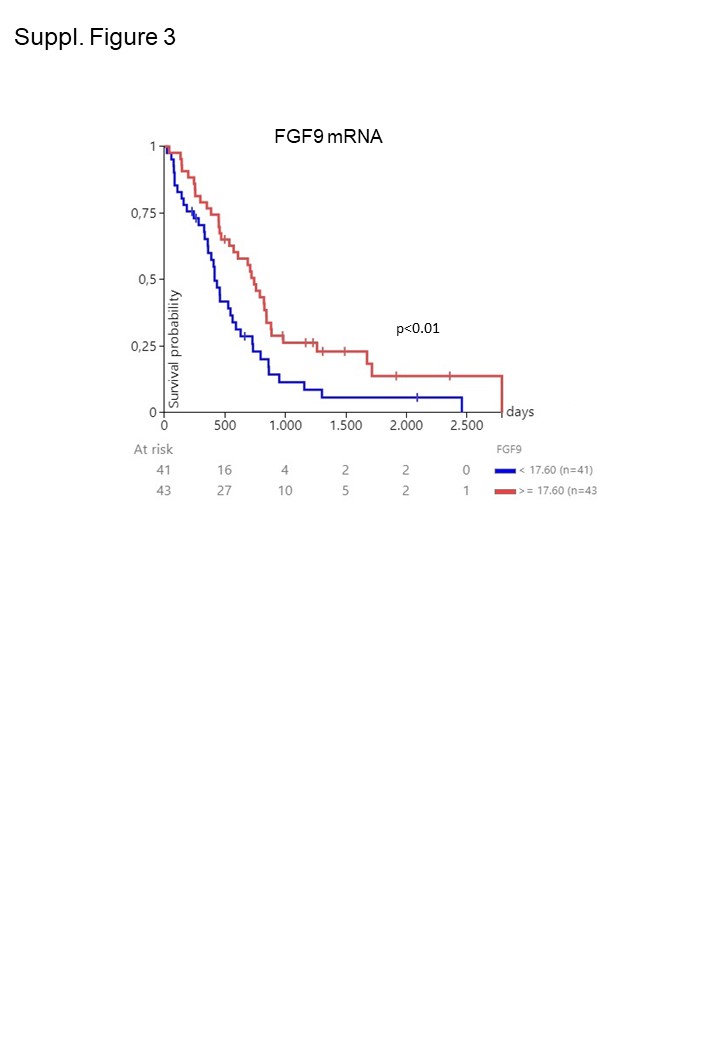

Supplement: Supplementary file 1 [file ijms-25-05355-s001.zip › Suppl. Fig 3.JPG]

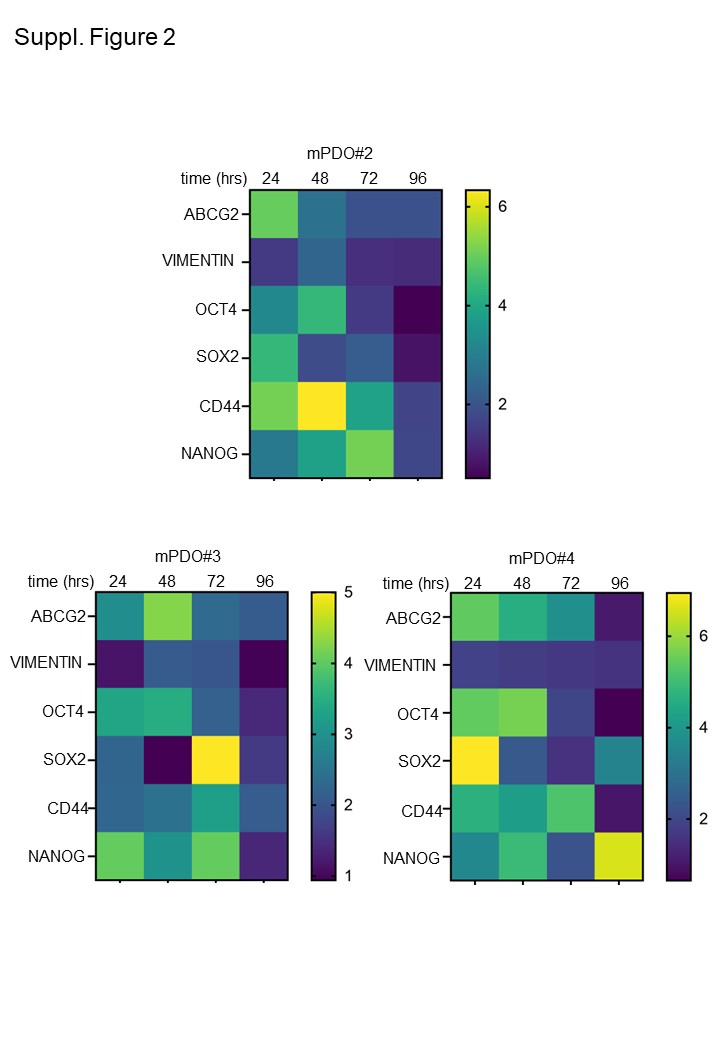

Supplement: Supplementary file 1 [file ijms-25-05355-s001.zip › Suppl.Fig 2.JPG]
